# Metabolic Network Analysis-Based Identification of Antimicrobial Drug Targets in Category A Bioterrorism Agents

**DOI:** 10.1371/journal.pone.0085195

**Published:** 2014-01-15

**Authors:** Yong-Yeol Ahn, Deok-Sun Lee, Henry Burd, William Blank, Vinayak Kapatral

**Affiliations:** 1 School of Informatics and Computing, Indiana University, Bloomington, Indiana, United States of America; 2 Department of Natural Medical Sciences and Department of Physics, Inha University, Incheon, Korea; 3 Igenbio.Inc, Chicago, Illinois, United States of America; Université Paris Descartes, INSERM, France

## Abstract

The 2001 anthrax mail attacks in the United States demonstrated the potential threat of bioterrorism, hence driving the need to develop sophisticated treatment and diagnostic protocols to counter biological warfare. Here, by performing flux balance analyses on the fully-annotated metabolic networks of multiple, whole genome-sequenced bacterial strains, we have identified a large number of metabolic enzymes as potential drug targets for each of the three Category A-designated bioterrorism agents including *Bacillus anthracis, Francisella tularensis and Yersinia pestis*. Nine metabolic enzymes- belonging to the coenzyme A, folate, phosphatidyl-ethanolamine and nucleic acid pathways common to all strains across the three distinct genera were identified as targets. Antimicrobial agents against some of these enzymes are available. Thus, a combination of cross species-specific antibiotics and common antimicrobials against shared targets may represent a useful combinatorial therapeutic approach against all Category A bioterrorism agents.

## Introduction

Bacterial Category A agents include *Bacillus anthracis* (anthrax), *Yersinia pestis* (bubonic plague), *Francisella tularensis* (tularemia), and the botulism toxin of *Clostridium botulinum* [1]. Despite the development of new, rapid methods for their identification, therapeutic and prophylactic challenges remain [2]. A systematic method for the analysis of multiple strains and a blueprint for antimicrobial discovery using genomics and computational tools for microbes have been recently described [3]. These computational approaches are highly cost effective and can be used to identify sets of targets across these biological warfare agents.

*Bacillus anthracis*, the Gram-positive agent causative of anthrax is naturally found in animals and in soil worldwide. They can survive both under aerobic and anaerobic conditions and can form heat resistant spores that make it an ideal agent for biological warfare. *B. anthracis* exhibits high genetic homogeneity, as determined by partial genome DNA microarray hybridization experiments and by genome sequencing. However, using variable number tandem repeats (VNTR) and multiple locus VNTR analyses researchers have identified genomic variation among diverse geographical isolates of *B. anthracis*. Based on historical, DNA analysis, SNP variations, molecular signatures and microbiological methods, they have been grouped into A, B, and C clusters and sub-lineages in each of the major clusters (Aβ, A1, A2, A3, A4, B1, B2 and C) [4]. Currently, antibiotics such as ciprofloxacin or deoxycycline are used both for prophylactic measure and for the treatment of anthrax patients, but currently there are no other targets or small molecules in the treatment pipeline.

*Francisella tularensis*, a Gram negative, facultative, intracellular mammalian pathogen, is a causative agent of zoonotic infections [5], [6]. It is found predominantly in the Northern hemisphere and Mediterranean parts of the world. Despite different geographical occurrence, the genome-wide sequence comparisons indicate only a limited genetic diversity of less than 4% among these species [7]. However, there is an extensive allelic variation due the presence of short sequences and tandem repeats [7], [8]. *F. tularensis* can be grouped into four distinct subspecies; *F. tularensis sub spp. tularensis*, *F. tularensis sub spp holartica*, and *F. tularensis sub spp mediasiatica*, and *F. tularensis spp novicida*. *F. tularensis* (Biovar type A) is highly virulent and occur predominantly in North America. *F. tularensis sub spp holarctica* (Biovar type B) is the primary cause of tularemia in Europe and is relatively non-pathogenic to humans [9]. Comparative virulence and pathogenic features due to large-scale sequence rearrangements among virulence species have been carefully identified [10], [11]. Except for *F. tularensis sub spp novicida*, there is no systematic identification of essential genes as targets for drug discovery among this group of bacteria [12]. Antibiotics against Gram-negative bacteria such as streptomycin or gentamycin are used as primary therapeutic choice, wheras doxycyline or ciprofloxacin are usually recommended for prophylactic treatment. However, the emergence of drug resistance or the intentional release of multi drug-resistant engineered strains is a potential threat to human life. Recent identification of erythromycin-resistant *F. tularensis sub spp holarctica* emphasizes the need for newer targets and drug identification. There is also an increased effort for human vaccine development, as the current *F. tularensis sub spp holarctica* LVS (Live Vaccine Strain) strain is ineffective in certain populations.

*Yersinia pestis*, the causative agent of the plague, is a Gram-negative enteric bacterium [13] that has caused one of the deadliest epidemics in human history in Europe in the 14^th^ century. *Y. pestis* is significantly diverse and is divided into three major branches, Branch 0 (Microtus and Pestiodes isolates), Branch 1 (Orientalis, African Antiqua), and Branch 2 (Medievalis and Asian isolates) [10]. Recently, Charusanti *et al* [14] have built a metabolic model and experimentally identified several potential drug targets of a clinical *Y. pestis* CO92 isolate. However, the sequence diversity among various *Y. pestis* geographical isolates is high, which is reflected in their metabolic capabilities and demonstrates the need for strain specific target identification. Despite the successful use of antibiotics such as streptomycin or gentamycin as primary therapeutic choice in the treatment of infection, doxycyline, ciprofloxacin or chloramphenicol is also administered as prophylactic drug of choice.

In the past few years, there has been a significant effort in genome sequencing and molecular diagnostics studies for diverse strains of the three Category A bioterrorism agents. Despite sequencing of diverse geographical isolates, there has been only very limited new target discovery and virtually no specific drug development. Identifying gene essentiality by experimental approaches either using transposon mutagenesis or RNA silencing is time consuming and expensive, and the results are strain-specific. In contrast, computational methods provide an alternate approach for the identification of single essential and synthetic lethal metabolic enzymes [10], [15], [16], [17], [18] that can be simultaneously tested for multiple strains [16], [19]. These methods can be also tested simultaneously under several growth conditions and identify organism/strain specific essential metabolic enzymes as common drug targets. Here, we have used these methods, as described earlier for multiple *S. aureus* strains [19] to identify genus specific and universally common metabolic enzyme as targets for *B. anthracis*, *F. tularensis*, and *Y. pestis*.

## Results

Bacterial genome variations are manifested in the metabolic or physiological characteristics of a given organism. Identification of these variations defines unique biochemical capabilities that allow growth and survival in a specific ecological niche. However, recognizing common core metabolic capabilities allow enzymatic target identification for anti-infective discovery. Using this logic and computational approaches for *E. coli* [15], [16] and for multiple strains of *S. aureus* [19] and in combination with classical molecular screening, we have identified small molecule inhibitors for *E. coli* and *S. aureus* [3]. Similarly, using these approaches and reactions associated with metabolic enzymes, we performed FBA for *B. anthracis*, *F. tularensis* and *Y. pestis* strains for common target identification.

We initially identified metabolic pathways, reactions and compounds for each of the strains within the same genus and then compared between the genera. On an average, the number of reactions is larger for both *B. anthracis* and *Y. pestis* compared to *F. tularensis*, which is attributed to the size of the genome, number of ORFs, etc. (Table 1). The transport reactions also varied among the three genera, although they were identical within the same genera. However, the number of metabolites varied among the strains of a given genera. After computing FBA and essentiality, the numbers of essential enzymes were in the range of 37–40 in *B. anthracis* and *Y. pestis* but twice as high in *F. tularensis* (the individual strain single essentiality data is provided in Table S1). Similarly, the essential metabolites from these essential reactions were higher in *F. tularensis* compared to *B. anthracis* and *Y. pestis* (Table 2). Based on these data we built networks of essential enzymes, reactions and compounds for *B. anthracis*, *Y. pestis*, and *F. tularensis* and the roles of these essential metabolic enzymes are described in the following sections.

**Table 1:** Genome features, with ORFs and functions, EC numbers, metabolic reactions, transport and metabolites of Category A bacterial agents.

Sl	Organism	Genome Code	NCBI Accession No	ORFs	ORFs with Functions	ORFs with EC no	Unique complete EC no	Very Likely Reactions	Likely Reactions	Cellular Reactions	Transport and exchange Reactions	Metabolites
1	*B. anthracis A1055*	BAA	PRJNA54131	5923	4771	1515	631	1122	132	1406	169	1820
2	*B. anthracis Ames ancestor*	BAH	PRJNA58083	5617	4502	1413	633	1123	132	1411	169	1824
3	*B. anthracis CNEVA-9066*	BAI	PRJNA54133	6123	4861	1525	635	1122	132	1410	169	1826
4	*B. anthracis Ames*	BAN	PRJNA57909	5311	4417	1392	634	1129	132	1415	169	1832
5	*B. anthracis Str Strene*	BAR	PRJNA58091	5287	4591	1488	635	1122	132	1410	169	1822
6	*B. anthracis Str Kruger B*	BAS	PRJNA54105	6190	4917	1545	634	1121	132	1409	169	1825
7	*B. anthracis A2012*	BAT	PRJNA54101	5662	4805	1550	640	1115	131	1410	169	1831
8	*B. anthracis Str Vollum*	BNT	PRJNA54135	6158	4881	1523	635	1122	132	1410	169	1826
1	*F. philomiragia (ATCC 25017)*	FPH	PRJNA39919	2022	1650	731	481	968	4	1129	113	1590
2	*F. tularensis sub spp. tularensis Schu S4*	FT	PRJNA57589	1722	1427	591	405	880	4	946	113	1423
3	*F. tularensis sub spp. mediasiactica*	FTC	PRJNA58939	1534	1226	564	387	816	3	876	113	1360
4	*F. tularensis sub spp. holarctica OSU18*	FTE	PRJNA32025	1705	1312	574	393	831	3	896	113	1372
5	*F. tularensis sub spp. novicida U112*	FTN	PRJNA58499	1801	1462	643	428	887	4	956	113	1407
6	*F. tularensis sub spp. tularensis FSC198*	FTR	PRJNA58693	1771	1428	595	411	911	4	980	113	1437
7	*F. tularensis sub spp. holarctica (LVS)*	FTU	PRJNA58595	1956	1604	648	428	942	4	1014	113	1452
1	*Y. pestis CO92*	YP	PRJNA57621	4066	3779	1143	660	1249	99	1444	208	1836
2	*Y. pestis Angola*	YPA	PRJNA58485	3925	3347	1080	643	1217	98	1411	208	1820
3	*Y. pestis biovar microtus str. 91001*	YPF	PRJNA58037	4142	3550	1118	651	1231	93	1420	208	1819
4	*Y. pestis KIM*	YPK	PRJNA15962	4201	3736	1180	663	1254	100	1458	208	1852

**Table 2:** Single essential enzymes and metabolites in the indicated strains identified using FBA for each of the strains studied.

	*Organism*	*Single essential Enzymes*	*Essential metabolites*
*1*	*B. anthracis A1055*	*38*	*81*
*2*	*B. anthracis Ames ancestor*	*39*	*81*
*3*	*B. anthracis CNEVA-9066*	*37*	*81*
*4*	*B. anthracis Ames*	*40*	*81*
*5*	*B. anthracis Str Strene*	*38*	*81*
*6*	*B. anthracis Str Kruger B*	*37*	*81*
*7*	*B. anthracis A2012*	*38*	*81*
*8*	*B. anthracis Str Vollum*	*37*	*81*
*1*	*F. tularensis sub spp. tularensis Schu S4*	*74*	*133*
*2*	*F. tularensis sub spp. mediasiactica*	*85*	*137*
*3*	*F. tularensis sub spp. holarctica OSU18*	*74*	*125*
*4*	*F. tularensis sub spp. novicida U112*	*69*	*121*
*5*	*F. tularensis sub spp. tularensis FSC198*	*80*	*120*
*6*	*F. tularensis sub spp. holarctica (LVS)*	*68*	*109*
*7*	*F. philomiragia (ATCC25017)*	*66*	*104*
*1*	*Y. pestis CO92*	*39*	*80*
*2*	*Y. pestis Angola*	*40*	*82*
*3*	*Y. pestis biovar microtus str. 91001*	*40*	*80*
*4*	*Y. pestis KIM*	*40*	*82*

### Identification of common metabolic essential enzymes in *B. anthracis*

**Figure 1:**
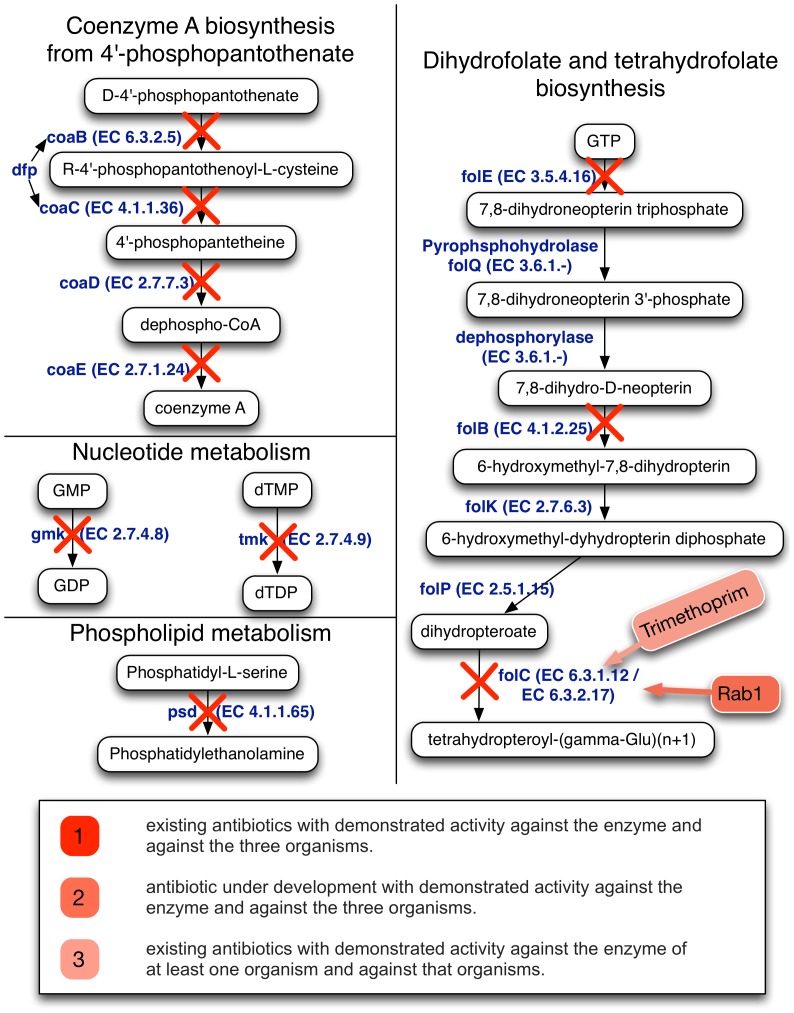
Common metabolic enzyme targets in the three Category A bacteria. Nine shared essential enzymes in several major pathways were identified across all Category A bacteria and are marked by “X”.

Using FBA methods, we have identified 35 metabolic enzymes that were calculated as essential for growth and biomass production in all *B. anthracis* strains. The enzymes and their associated biochemical reactions are given in Table 3. The majority of the targets are involved in the amino-acids, vitamins, nucleotides or cofactors biosynthesis pathways (Fig. 1).

**Table 3:** Essential enzymes and its associated reactions identified by FBA are categorized into specific metabolic systems of Category A bacteria. B: *B. anthracis*, F: *F. tularensis*, Y: *Y. pestis.*

Sl	Genomes	Gene	EC#	Functions	Reactions
**Amino acid biosynthesis**					
1	B	*hisD*	1.1.1.23	Histidinol dehydrogenase	L-histidinol+H(2)O+2 NAD(+) < = > L-histidine+2 NADH
2	B	*hisG*	2.4.2.17	ATP phosphoribosyltransferase	1-(5-phospho-D-ribosyl)-ATP+diphosphate < = > ATP+5-phospho-alpha-D-ribose 1-diphosphate
3	B	*hisB*	4.2.1.19	Imidazoleglycerol-phosphate dehydratase	D-erythro-1-(imidazol-4-yl)glycerol 3-phosphate < = > 3-(imidazol-4-yl)-2-oxopropyl phosphate+H(2)O
4	B	*hisA*	5.3.1.16	1-(5-phosphoribosyl)-5-[(5-phosphoribosylamino)methylideneamino] imidazole-4-carboxamide isomerase	1-(5-phosphoribosyl)-5-((5-phosphoribosylamino)methylideneamino)imidazole-4-carboxamide < = > 5-((5-phospho-1-deoxyribulos-1-ylamino)methylideneamino)-1-(5-phosphoribosyl)imidazole-4-carboxamide
5	B,Y	*Meth*	2.1.1.13	5-methyltetrahydrofolate-homocysteine methyltransferase homocysteine-binding subunit	5-methyltetrahydrofolate+NAD(P)(+) < = > 5,10-methylenetetrahydrofolate+NAD(P)H
6	B	*metF*	1.5.1.20	Methylenetetrahydrofolate reductase	5-methyltetrahydrofolate+L-homocysteine < = > tetrahydrofolate+L-methionine
7	F,Y	*metK*	2.5.1.6	Methionine adenosyltransferase	ATP+L-methionine+H(2)O < = > phosphate+diphosphate+S-adenosyl-L-methionine
8	Y	*mtnN*	3.2.2.16/3.2.2.9	Methylthioadenosine nucleosidaseAdenosylhomocysteine nucleosidase	S-methyl-5′-thioadenosine+H(2)O < = > S-methyl-5-thio-D-ribose+adenineS-adenosyl-L-homocysteine+H(2)O < = > S-(5-deoxy-D-ribos-5-yl)-L-homocysteine+adenine
9	B	*trpD*	2.4.2.18	Anthranilate phosphoribosyltransferase	N-(5-phospho-D-ribosyl)-anthranilate+diphosphate < = > anthranilate+5-phospho-alpha-D-ribose 1-diphosphate
10	B	*trpC*	4.1.1.48	Indole-3-glycerol-phosphate synthase	1-(2-carboxyphenylamino)-1-deoxy-D-ribulose 5-phosphate < = > 1-C-(3-indolyl)-glycerol 3-phosphate+CO(2)+H(2)O
11	B	*trpF*	5.3.1.24	Phosphoribosylanthranilate isomerase	N-(5-phospho-beta-D-ribosyl)anthranilate < = > 1-(2-carboxyphenylamino)-1-deoxy-D-ribulose 5-phosphate
12	B	*dapH*	2.3.1.89	Tetrahydrodipicolinate N-acetyltransferase	Acetyl-CoA+(S)-2,3,4,5-tetrahydropyridine-2,6-dicarboxylate+H(2)O < = > CoA+L-2-acetamido-6-oxoheptanedioate
13	B	*dapB*	1.3.1.26	Dihydrodipicolinate reductase	(S)-2,3,4,5-tetrahydrodipicolinate+NAD(P)(+) < = > (S)-2,3-dihydrodipicolinate+NAD(P)H
14	B	*dapL*	3.5.1.47	N-acetyldiaminopimelate deacetylase	N-acetyl-LL-2,6-diaminoheptanedioate+H(2)O < = > acetate+LL-2,6-diaminoheptanedioate
15	B, Y	*dapF*	5.1.1.7	Diaminopimelate epimerase	LL-2,6-diaminoheptanedioate < = > meso-diaminoheptanedioate
16	B,Y	*lysA*	4.1.1.20	Diaminopimelate decarboxylase	Meso-2,6-diaminoheptanedioate < = > L-lysine+CO(2)
17	B	*aroA/aroH*	5.4.99.5/2.5.1.54	Chorismate mutase/3-deoxy-7-phosphoheptulonate synthase	Chorismate < = > prephenate Phosphoenolpyruvate+D-erythrose 4-phosphate+H(2)O < = > 3-deoxy-D-arabino-hept-2-ulosonate 7-phosphate+phosphate
18	Y	*speE*	2.5.1.16	Spermidine synthase	S-Adenosylmethioninamine+Putrescine < = > 5′-Methylthioadenosine+Spermidine
19	F	*gltX1*	6.1.1.17	Glutamate-tRNA ligase	ATP+L-glutamate+tRNA(Glu) < = > AMP+diphosphate+L-glutamyl-tRNA(Glu)
20	Y	*sped*	4.1.1.50	Adenosylmethionine decarboxylase	S-adenosyl-L-methionine < = > (5-deoxy-5-adenosyl)(3-aminopropyl)-methylsulfonium salt+CO(2)
21	B	*aroH*	2.5.1.54	3-deoxy-7-phosphoheptulonate synthase	Phosphoenolpyruvate+D-erythrose 4-phosphate+H(2)O < = > 3-deoxy-D-arabino-hept-2-ulosonate 7-phosphate+phosphate
22	B	*aroA*	5.4.99.5	Chorismate mutase	Chorismate < = > prephenate
**Vitamins and cofactors biosynthesis**					
23	B,Y	*pabC*	4.1.3.38	Aminodeoxychorismate lyase	4-amino-4-deoxychorismate < = > 4-aminobenzoate+pyruvate
24	B	*dfrA*	1.5.1.3	Dihydrofolate reductase	5,6,7,8-tetrahydrofolate+NADP(+) < = > 7,8-dihydrofolate+NADPH
25	B,Y	*folP*	2.5.1.15	Dihydropteroate synthase	2-amino-4-hydroxy-7,8-dihydropteridin-6-yl)methyl diphosphate+4-aminobenzoate < = > diphosphate+dihydropteroate
26	B,F,Y	*folE*	3.5.4.16	GTP cyclohydrolase I	GTP+H(2)O < = > formate+2-amino-4-hydroxy-6-(erythro-1,2,3-trihydroxypropyl)-dihydropteridine triphosphate
27	B,F,Y	*folB*	4.1.2.25	Dihydroneopterin aldolase	2-amino-4-hydroxy-6-(D-erythro-1,2,3-trihydroxypropyl)-7,8-dihydropteridine < = > 2-amino-4-hydroxy-6-hydroxymethyl-7,8-dihydropteridine+glycolaldehyde
28	B,F,Y	*folC*	6.3.2.12	Dihydrofolate synthase	ATP+7,8-dihydropteroate+L-glutamate < = > ADP+phosphate+7,8-dihydropteroylglutamate ATP+tetrahydropteroyl-(gamma-Glu)(n)+L-glutamate < = > ADP+phosphate+tetrahydropteroyl-(gamma-Glu)(n+1)
29	B,F,Y	*coaE*	2.7.1.24	Dephospho-CoA kinase	ATP+3′-dephospho-CoA < = > ADP+CoA
30	Y	*coaA*	2.7.1.33	Pantothenate kinase	ATP+(R)-pantothenate < = > ADP+(R)-4′-phosphopantothenate
31	B,F,Y	*coaD*	2.7.7.3	Pantetheine-phosphate adenylyltransferase	ATP+pantetheine 4′-phosphate < = > diphosphate+3′-dephospho-CoA
32	B	*nadD*	2.7.7.18	Nicotinate-nucleotide adenylyltransferase	ATP+nicotinate ribonucleotide < = > diphosphate+deamido-NAD(+)
33	F	*nadV*	2.4.2.12	Nicotinamide phosphoribosyltransferase	Nicotinamide D-ribonucleotide+diphosphate < = > nicotinamide+5-phospho-alpha-D-ribose 1-diphosphate
34	F,Y	*ppnK*	2.7.1.23	NAD(+) kinase	ATP+NAD(+) < = > ADP+NADP(+)
35	B,F,Y	*Dfp (coaB and coaC)*	4.1.1.36/6.3.2.5	Phosphopantothenoyl-cysteine decarboxylase Phosphopantothenate-cysteine ligase	N-((R)-4′-phosphopantothenoyl)-L-cysteine < = > pantotheine 4′-phosphate+CO(2)CTP+(R)-4′-phosphopantothenate+L-cysteine < = > CMP+PPi+N-((R)-4′-phosphopantothenoyl)-L-cysteine
36	F	*hemE*	4.1.1.37	Uroporphyrinogen decarboxylase	Uroporphyrinogen III < = > coproporphyrinogen+4 CO(2)
37	F	*hemC*	2.5.1.61	Hydroxymethylbilane synthase	4 porphobilinogen+H(2)O < = > hydroxymethylbilane+4 NH(3)
38	F	*hemF*	1.3.3.3	Coproporphyrinogen oxidase	Coproporphyrinogen-III+O(2)+2 H(+) < = > protoporphyrinogen-IX+2 CO(2)+2 H(2)O
39	F	*hemB*	4.2.1.24	Porphobilinogen synthase	2 5-aminolevulinate < = > porphobilinogen+2 H(2)O
40	F	*hemD*	4.2.1.75	Uroporphyrinogen-III synthase	Hydroxymethylbilane < = > uroporphyrinogen III+H(2)O
41	F	*hemH*	4.99.1.1	Ferrochelatase	Protoheme+2 H(+) < = > protoporphyrin+Fe(2+)
42	F	*hemL*	5.4.3.8	Glutamate-1-semialdehyde 2,1-aminomutase	(S)-4-amino-5-oxopentanoate < = > 5-aminolevulinate
43	F,Y	*ribC (ribF)*	2.7.1.26/2.7.7.2	Riboflavin kinaseFAD synthetase	ATP+riboflavin < = > ADP+FMNATP+FMN < = > diphosphate+FAD
44	Y	*ribA*	3.5.4.25	GTP cyclohydrolase II	GTP+3 H(2)O < = > formate+2,5-diamino-6-hydroxy-4-(5-phospho-D-ribosylamino)pyrimidine+diphosphate
45	F,Y	*ribD*	1.1.1.193/3.5.4.26	5-amino-6-(5-phosphoribosylamino)uracil reductase Diaminohydroxyphosphoribosylaminopyrimidine deaminase	5-amino-6-(5-phospho-D-ribitylamino)uracil+NADP(+) < = > 5-amino-6-(5-phospho-D-ribosylamino)uracil+NADPH2,5-diamino-6-hydroxy-4-(5-phosphoribosylamino)pyrimidine+H(2)O < = > 5-amino-6-(5-phosphoribosylamino)uracil+NH(3)
46	F	*ubiC*	4.1.3.40	Chorismate lyase	Chorismate < = > 4-hydroxybenzoate+pyruvate
**Non-mevalonate pathway for isoprenoid biosynthesis**					
47	F	*ispH*	1.17.1.2	4-hydroxy-3-methylbut-2-enyl diphosphate reductase	Isopentenyl diphosphate+NAD(P)(+)+H(2)O < = > (E)-4-hydroxy-3-methylbut-2-en-1-yl diphosphate+NAD(P)HDimethylallyl diphosphate+NAD(P)(+)+H(2)O < = > (E)-4-hydroxy-3-methylbut-2-en-1-yl diphosphate+NAD(P)H
48	F	*ispD*	2.7.7.60	2-C-methyl-D-erythritol 4-phosphate cytidylyltransferase	CTP+2-C-methyl-D-erythritol 4-phosphate < = > diphosphate+4-(cytidine 5′-diphospho)-2-C-methyl-D-erythritol
49	F	*ispF*	4.6.1.12	2-C-methyl-D-erythritol 2,4-cyclodiphosphate synthase	2-phospho-4-(cytidine 5′-diphospho)-2-C-methyl-D-erythritol < = > 2-C-methyl-D-erythritol 2,4-cyclodiphosphate+CMP
50	F	*Dxs*	2.2.1.7	1-deoxy-D-xylulose-5-phosphate synthase	Pyruvate+D-glyceraldehyde 3-phosphate < = > 1-deoxy-D-xylulose 5-phosphate+CO(2)
51	F	*uppS*	2.5.1.31	Di-trans,poly-cis-undecaprenyl-diphosphate synthase ((2E,6E)-farnesyl- diphosphate specific)	(2E,6E)-farnesyl diphosphate+8 isopentenyl diphosphate < = > 8 diphosphate+di-trans,octa-cis-undecaprenyl diphosphate
52	F	*ispE*	2.7.1.148	4-(cytidine 5′-diphospho)-2-C-methyl-D-erythritol kinase	ATP+4-(cytidine 5′-diphospho)-2-C-methyl-D-erythritol < = > ADP+2-phospho-4-(cytidine 5′-diphospho)-2-C-methyl-D-erythritol
53	F	*Dxr*	1.1.1.267	1-deoxy-D-xylulose-5-phosphate reductoisomerase	2-C-methyl-D-erythritol 4-phosphate+NADP(+) < = > 1-deoxy-D-xylulose 5-phosphate+NADPH
**Nucleotides biosynthesis**					
54	B,F,Y	*Gmk*	2.7.4.8	Guanylate kinase	ATP+GMP < = > ADP+GDP
55	B,F,Y	*Tmk*	2.7.4.9	dTMP kinase	ATP+dTMP < = > ADP+dTDP
56	F	*deoD*	2.4.2.1	Purine-nucleoside phosphorylase	Purine nucleoside+phosphate < = > purine+alpha-D-ribose 1-phosphate
**Fatty acid metabolism**					
57	B	*fadB*	1.1.1.35/4.2.1.17/5.3.3.8	Enoyl-CoA hydratase Delta(3) -cis-delta(2,3-hydroxyacyl-CoA dehydrogenase -trans-enoyl-CoA isomerase	S)-3-hydroxyacyl-CoA+NAD(+) < = > 3-oxoacyl-CoA+NADH(3S)-3-hydroxyacyl-CoA < = > trans-2(or 3)-enoyl-CoA+H(2)O(3Z)-dodec-3-enoyl-CoA < = > (2E)-dodec-2-enoyl-CoA
58	B	*fadA*	2.3.1.16	3-ketoacyl-CoA thiolase	Acyl-CoA+acetyl-CoA < = > CoA+3-oxoacyl-CoA
**Phospholipid biosynthesis**					
59	B	*ugtP*	2.4.1.-2.4.1.157	Glucosyldiacylglycerol 6-beta-glucosyltransferase 1,2-diacylglycerol 3-glucosyltransferase	UDP-glucose+1,2-diacyl-sn-glycerol < = > UDP+3-D-glucosyl-1,2-diacyl-sn-glycerol3-D-Glucosyl-1,2-diacylglycerol+UDP-glucose < = > beta-D-glucosyl-1,6-beta-D-glucosyl-1,3-diacylglycerol+UDP
60	B,F	*cdsA*	2.7.7.41	Phosphatidate cytidylyltransferase	CTP+phosphatidate < = > diphosphate+CDP-diacylglycerol
61	B,F,Y	*psd*	4.1.1.65	Phosphatidylserine decarboxylase	Phosphatidyl-L-serine < = > phosphatidylethanolamine+CO(2)
62	Y,F	*pssA*	2.7.8.8	CDP-diacylglycerol–serine O-phosphatidyltransferase	CDP-diacylglycerol+L-serine < = > CMP+(3-sn-phosphatidyl)-L-serine
**Cell wall biosynthesis**					
63	B,F	*mraY*	2.7.8.13	Phospho-N-acetylmuramoyl-pentapeptide-transferase	UDP-Mur2Ac (oyl-L-Ala-gamma-D-Glu-L-Lys-D-Ala-D-Ala)+undecaprenyl phosphate < = > UMP+Mur2Ac(oyl-L-Ala-gamma-D-Glu-L-Lys-D-Ala-D-Ala)-diphosphoundecaprenol
64	B	*glum*	2.3.1.157/2.7.7.23	Glucosamine-1-phosphate acetyltransferase UDP-N-acetylglucosamine pyrophosphorylase	Acetyl-CoA+alpha-D-glucosamine 1-phosphate < = > CoA+N-acetyl-alpha-D-glucosamine 1-phosphateUTP+N-acetyl-alpha-D-glucosamine 1-phosphate < = > diphosphate+UDP-N-acetyl-D-glucosamine
65	B	*gale*	5.1.3.2/5.1.3.7	UDP-glucose 4-epimerase UDP-N-acetylglucosamine 4-epimerase	UDP-glucose < = > UDP-galactoseUDP-N-acetyl-D-glucosamine < = > UDP-N-acetyl-D-galactosamine
66	B,F	*glmM*	5.4.2.10	Phosphoglucosamine mutase	Alpha-D-glucosamine 1-phosphate < = > D-glucosamine 6-phosphate
67	F,Y	*lpxA*	2.3.1.129	Acyl-[acyl-carrier-protein]–UDP-N-acetylglucosamine O-acyltransferase	R)-3-hydroxytetradecanoyl-[acyl-carrier-protein]+UDP-N-acetylglucosamine < = > [acyl-carrier-protein]+UDP-3-O-(3-hydroxytetradecanoyl)-N-acetylglucosamine
68	F,Y	*lpxB*	2.4.1.182	Lipid-A-disaccharide synthase	UDP-2,3-bis(3-hydroxytetradecanoyl)glucosamine+2,3-bis(3-hydroxytetradecanoyl)-beta-D-glucosaminyl 1-phosphate < = > UDP+2,3-bis(3-hydroxytetradecanoyl)-D-glucosaminyl-1,6-beta-D-2,3-bis(3-hydroxytetradecanoyl)-beta-D-glucosaminyl 1-phosphate
69	F	*murG*	2.4.1.227	Undecaprenyldiphosphomuramoylpentapeptide beta-N- acetylglucosaminyltransferase	UDP-N-acetylglucosamine+Mur2Ac(oyl-L-Ala-gamma-D-Glu-L-Lys-D-Ala-D-Ala)-diphosphoundecaprenol < = > UDP+GlcNAc-(1→4)-Mur2Ac(oyl-L-Ala-gamma-D-Glu-L-Lys-D-Ala-D-Ala)-diphosphoundecaprenol
70	F	*murB*	1.1.1.158	UDP-N-acetylmuramate dehydrogenase	UDP-N-acetylmuramate+NADP(+) < = > UDP-N-acetyl-3-O-(1-carboxyvinyl)-D-glucosamine+NADPH
71	F,Y	*kdsA*	2.5.1.55	3-deoxy-8-phosphooctulonate synthase	Phosphoenolpyruvate+D-arabinose 5-phosphate+H(2)O < = > 2-dehydro-3-deoxy-D-octonate 8-phosphate+phosphate
72	F	*murA*	2.5.1.7	UDP-N-acetylglucosamine 1-carboxyvinyltransferase	Phosphoenolpyruvate+UDP-N-acetyl-D-glucosamine < = > phosphate+UDP-N-acetyl-3-O-(1-carboxyvinyl)-D-glucosamine
73	F,Y	*lpxK*	2.7.1.130	Tetraacyldisaccharide 4′-kinase	ATP+(2-N,3-O-bis(3-hydroxytetradecanoyl)-beta-D-glucosaminyl)-(1→6)-(2-N,3-O-bis(3-hydroxytetradecanoyl)-beta-D-glucosaminyl phosphate) < = > ADP+(2-N,3-O-bis(3-hydroxytetradecanoyl)-4-O-phosphono-beta-D-glucosaminyl)-(1→6)-(2-N,3-O-bis(3-hydroxytetradecanoyl)-beta-D-glucosaminyl phosphate)
74	F,Y	*kdsB*	2.7.7.38	3-deoxy-manno-octulosonate cytidylyltransferase	CTP+3-deoxy-D-manno-octulosonate < = > diphosphate+CMP-3-deoxy-D-manno-octulosonate
75	F	*murF*	6.3.2.10	UDP-N-acetylmuramoyl-tripeptide–D-alanyl-D-alanine ligase	ATP+UDP-N-acetylmuramoyl-L-alanyl-gamma-D-glutamyl-L-lysine+D-alanyl-D-alanine < = > ADP+phosphate+UDP-N-acetylmuramoyl-L-alanyl-gamma-D-glutamyl-L-lysyl-D-alanyl-D-alanine
76	F	*murD*	6.3.2.9	UDP-N-acetylmuramoyl-L-alanine–D-glutamate ligase	ATP+UDP-N-acetylmuramoyl-L-alanine+glutamate < = > ADP+phosphate+UDP-N-acetylmuramoyl-L-alanyl-D-glutamate
77	F	*murI*	5.1.1.3	Glutamate racemase	L-glutamate < = > D-glutamate
78	Y	*kdsC*	3.1.3.45	3-deoxy-manno-octulosonate-8-phosphatase	3-deoxy-D-manno-octulosonate 8-phosphate+H(2)O < = > 3-deoxy-D-manno-octulosonate+phosphate
79	Y	*hldD*	5.1.3.20	ADP-glycero-manno-heptose 6-epimerase	ADP-D-glycero-D-manno-heptose < = > ADP-L-glycero-D-manno-heptose
**Carbohydrate metabolism**					
80	Y	*glgA*	2.4.1.21	Starch synthase	ADP-glucose+(1,4-alpha-D-glucosyl)(n) < = > ADP+(1,4-alpha-D-glucosyl)(n+1)
81	Y	*rhaB*	2.7.1.5	Rhamnulokinase	ATP+L-rhamnulose < = > ADP+L-rhamnulose 1-phosphate
82	Y	*lyx*	2.7.1.53	L-xylulokinase	ATP+L-xylulose < = > ADP+L-xylulose 5-phosphate
83	Y	*ksdD*	5.3.1.13	Arabinose-5-phosphate isomerase	D-Arabinose 5-phosphate < = > D-Ribulose 5-phosphate

Enzymes involved in the biosynthesis of L-histidine from 5-phospho-α D-ribosyl 1-diphosphate and ATP such as HisD, HisG, HisB and HisA are essential (Table 3). 5-phospho-α D-ribosyl 1-diphosphate is precursor both for histidine and IMP biosynthetic pathway and is involved in 5′-phosphoribosyl-4-4carboxamide-5-aminoimidazole (AICAR) cycle. Enzymes involved in L-methionine biosynthesis (MetH, MetF) were found to be essential as L-methionine is required for a number of cellular functions, including initiation of protein synthesis, the methylation of DNA, rRNA and the biosynthesis of cysteine, phospholipids and polyamines. Enzymes involved in the L-tryptophan biosynthesis (TrpD, TrpC, and TrpF) using chorismate as precursor are essential as tryptophan is precursor of indole in many bacteria. Pathways for the synthesis for L-lysine and LL-diaminopimelate from L-aspartate catalyzed by enzymes encoded by *dapH*, *dapB*, *dapL*, *lysA*, and *dapF* were determined to be essential. Diaminopimelate is used for both the biosynthesis of lysine and peptidoglycan. Other enzymes involved in L-phenylalanine and L-tyrosine biosyntheses (AroA, AroH) were also identified as essential in *B. anthracis* (Table 3).

Among vitamins, enzymes involved in the folate biosynthesis pathway, enzymes such as PabC DfrA, FolB, FolC, FolE and FolP were identified to be essential. Other enzymes involved in cofactors synthesis such as coenzyme A biosynthesis (CoaE, CoaD), pantothenate (Dfp) and *de novo* biosynthesis/salvage of NAD and NADPH (NadD) were also identified as essential. Only two enzymes involved in the purine (guanosine) nucleotide biosynthesis (guanylate kinase Gmk) and thymidine nucleotide biosynthesis (dTMP kinase, Tmk) were identified as essential for biomass production.

In lipid metabolism, enzymes involved in the glycerolipid (diglucosyldiacyl glycerol) biosynthesis (UgtP) that catalyzes the formation of mono-, di- and triglucosyldiacyl glycerol were identified as essential. Diglucosyldiacylglycerol is a predominant glycolipid used as a membrane anchor for lipoteichoic acid. A second essential enzyme, cytidylyl-transferase (CdsA), which is involved in CDP-diglyceride biosynthesis, a major component for phosphatidyl group of phospholipids was also identified as essential. Another key enzyme, phosphatidylserine decarboxylase (Psd) involved in phospholipid (phosphatidylethanolamine) biosynthesis was found to be essential. Phosphoethanolamine head groups of phosphatidylethanolamine are transferred and attached to the LPS core sugars and to periplasmic membrane-derived oligosaccharides. Among the enzymes involved in cell wall biosynthesis, phospho-N-acetylmuramoyl-pentapeptide-transferase (MraY) involved in peptidoglycan biosynthesis is essential, as in *E. coli* [20]. A second enzyme, glucosamine-1-phosphate acetyltransferase (GlmU), is involved in UDP-*N*-acetyl-D-glucosamine biosynthesis, an essential precursor of peptidoglycan. A third UDP-glucose 4-epimerase (GalE), which is involved in galactose, amino sugar and nucleotide sugar metabolism, was also identified as essential enzyme in the cell wall biosynthesis. UDP-D-galactose is a building block for colonic acid and mycolyl-arabinogalactan-peptidoglycan complex biosynthesis. Phosphoglucosamine mutase (GlmM) involved in cell-wall peptidoglycan and LPS biosyntheses was determined to be essential. It is interesting to note that enzymes involved in fatty acid metabolism, specifically in β-oxidation (FadA, FadB) are essential for growth. This conservation implies that the β-oxidation of fatty acids has an indispensable function under certain physiological conditions. In fact, the *fadNA-E* operon encoding the β-oxidation catalyzing enzymes is induced at the onset of sporulation. This induction requires the *yvbA* protein involved in cannibalism by sporulating cells [21]. None of fatty acid biosynthetic enzymes (FabA, FabB, FabI, FabG etc.) were found to be essential under the conditions tested.

### Identification of common metabolic essential enzymes in *F. tularensis*

Although the genome wide sequence variations of *Francisella* species are in the order of 3–5%, there is a significant variation in the number of unique enzymes and metabolic reactions among the *F. tularensis* sub species. The single essential enzymes for individual strains of the diverse isolates of *F. tularensis* are given in Table S1. We identified a total of 46 single essential enzymes across the seven species, the majority of which were identified in the vitamins, cofactors, and cell wall biosynthesis pathways.

Unlike in *B. anthracis*, fewer enzymes in amino acid biosynthesis were identified as essential for growth in *F. tularensis*. These include methionine adenosyltransferase (MetK) involved in cysteine/methionine metabolism, which has been shown to be essential for the growth of *E. coli* K-12 [22]. An enzyme involved in lysine biosynthesis (MurF), which is required for the synthesis of peptidoglycan, is also essential. In the glutamate sub-system, ORFs for tRNA-dependent L-glutamate biosynthesis (*gltX1*) and L-glutaminyl-tRNA were determined as essential under the conditions tested (Table 3, Fig. 1). Several enzymes in the vitamin subsystem involved in the biosynthesis of porphyrin, heme and tetrapyrrole (HemF, HemC, HemE, HemB, HemD, HemH, and HemL1) were identified as essential.

In all the *F. tularensis sub species*, two metabolic routes for NAD synthesis has been identified [23], but most of the enzymes involved in the NAD biosynthesis pathway were identified as non-essential in our approaches except for NAD kinase, a key step in the phosphorylation of NAD to form NADP. Neither NMN synthetase nor NAD synthetases were identified to be essential in our study. Three enzymes belonging to the coenzyme A biosynthesis pathway (CoaE, CoaD and CoaBC) and riboflavin (FMN) biosynthesis (RibC, RibF and RibD) and folate biosynthesis pathway (FolE, FolB, and FolC), nicotinate/nicotinamide biosynthesis (PpnK) and ubiquinone biosynthesis pathway (UbiC) were identified. Isoprenoids necessary for ubiquinone production are synthesized using the non-mevalonate pathway in *F. tularensis*, and we identified several enzymes such as IspH, IspD, IspF, Dxs, UppS, IspE and Dxr as essential (Table 3). The intermediate metabolite undecaprenyl diphosphate is also a precursor of glycosyl carrier lipid, which is involved in the biosynthesis of bacterial cell wall polysaccharide components such as peptidoglycan and lipopolysaccharide. Only three enzymes in the phospholipid biosynthesis pathway (CdsA, PssA, and Psd) were identified as essential for biomass production.

Several enzymes involved in the synthesis of cell wall were identified to be essential among all the *F. tularensis* sub species. These include lipid A and peptidoglycan biosynthesis pathways and enzymes such as LpxA, LpxB, MurG, MurB, KdsA, MurA, LpxK, KdsB, MraY, MurF, MurD, MurI, and GlmM (Table 3). In purine and pyrimidine metabolism (synthesis, degradation and salvage) thymidine kinase (TmK), which is involved in the formation of dTDP using thymidine and is a well-known target for host cells that are infected with herpes virus, was identified. Other enzymes involved in nucleotides and deoxynucleotides metabolism such as guanylate kinase (GmK), purine nucleoside phosphorylase (DeoD) were identified as essential. Guanylate kinase converts GMP to GDP using ATP for the synthesis of nucleotide diphosphates such as ADP and GDP. dTMP kinase converts dTMP to dTDP using ATP (see Fig. 1).

### Identification of common essential metabolic enzymes in *Y. pestis*

Among the four *Y. pestis* genomes analyzed in this study, 37 single essential metabolic enzymes were common to all the four genomes (Table 3). The majority of these enzymes represented vitamins, cofactors, cell wall biosynthesis and very few were in amino-acid and carbohydrate pathways. We found 24 enzymes that are calculated to be essential in our study and that have been experimentally identified as essential in *Y. pestis* CO92 strain [14]. In contrast, nine enzymes (MetH, RhaB, Lyx, KdsC, MtnN, LysA, DapF, HidD and KdsD) were identified as essential in our study, but were found to be dispensable or non-essential in the *Y. pestis* CO92 strain [14].

The enzymes identified as essential by computational approaches were in the cofactor pathways including folate (FolP, FolE, FolB, FolC and PabC), coenzyme A (CoaB, CoaC, CoaD and CoaE), riboflavin (RibA, RibC and RibD) (FMN) biosynthesis, pantothenate biosynthesis (Dfp) and nicotinate/nicotinamide biosynthesis (PpnK). Seven enzymes involved in the lipid A and peptidoglycan biosynthesis was identified (LpxA, LpxB, LpxK, KdsA, KdsC, KdsB, KdsD and HldD). The specific reactions associated with these enzymes are given in Table 3. In addition, two genes, *gmK* (nucleotide biosynthesis) and *tmK* (thymidine) nucleotides biosynthesis was also identified as essential in all the *Y. pestis* genomes. Surprisingly, only two enzymes, phosphatidylserine decarboxylases (Psd) and CDP-diacylglycerol-serine O-phosphatidyltransferase (PssA) were determined as essential for growth.

Three enzymes in the carbohydrate metabolism, starch synthase (GlgA) involved in bacterial glycogen, ramnulokinase (RhaB) involved in pentose degradation, and L-xylulokinase (Lyx) involved in the breakdown of pentose sugars such as L-lyxose and L-xylulose were identified as essential for biomass and growth in *Y. pestis*. These enzymes may have evolved to play a specific role in metabolism in insect or human hosts under specific conditions. Finally, arabinose 5-phosphate isomerase (KdsD), which is involved in the synthesis of ribulose −5 phosphate that is necessary for nucleotides, was also identified as essential.

### Common targets among all Category A bioterrorism agents

Taken together, we have identified nine metabolic enzymes as being essential in all 19 strains spanning three genera of the three Category A bioterrorism agents (Table 4, Fig. 1). We then compared these common essential enzymes with experimentally validated essentiality data from other organisms using the DEG 5.0 database (http://tubic.tju.edu.cn/deg/) [24]. These common essentials belong to the cofactor synthesis pathway, including the Coenzyme A biosynthesis (phosphopantothenoyl cysteine decarboxylase (CoaB), phosphopantothenate cysteine ligase (CoaC), pantetheine-phosphate adenylyltransferase (CoaD), dephospho-CoA kinase (CoaE)) and folate biosynthesis pathways (dihydroneopterin aldolase (FolB), dihydrofolate synthase/tetrahydrofolate synthase (FolC), GTP cyclohydrolase I (FolE)) (Fig. 1). CoaE is essential for growth in *E. coli* [25], [26] and six other bacterial species (Table 4). CoaD was experimentally determined as essential in other pathogenic bacteria such as *V. cholera, H. influenzae, S. pneumoniae*, and others. Phosphatidyl serine decarboxylase (Psd), which is involved in the synthesis of phosphatidyl-ethanolamine was also found to be essential in other Gram-negative bacteria, including *F. tularensis sub spp novicida*, *E. coli*, and *S. enterica* (Table 4). Two other enzymes, guanylate kinase (Gmk) and thymidylate kinase (Tmk) belonging to the nucleic acid pathways, were experimentally found to be essential in *E. coli, B. subtilis, H.influenzae* and other bacteria [26], including *F. tularensis sub spp novicida* [12] and *Y. pestis* [14].

**Table 4:** Comparisons of predicted essential enzymes encoding genes shared by all three Category A agents to experimentally identified essential genes across various pathogenic bacteria.

Sl	Enzyme	Gene	Vc	Bs	Ec	Hi	Sp	Fn	Ab	Pa	Se	Sa	Mg	Mt	Mp	Hp
1	4.1.1.36/6.3.2.5	*Dfp (coaB, coaC)*	−	−	+	−	+	+	+	−	+	−	−	+	−	−
2	2.7.7.3	*coaD*	+	−	+	+	+	+	+	+	+	+	−	−	−	−
3	2.7.1.24	*coaE*	+	−	+	+	−	−	−	+	+	+	−	+	−	−
4	4.1.2.25	*folB*	−	−	−	−	−	+	+	+	+	−	−	+	−	+
5	6.3.2.12/6.3.2.17	*folC*	−	−	+	−	−	−	+	−	+	−	−	+	−	−
6	3.5.4.16	*folE*	+	−	+	−	−	−	+	−	+	−	−	+	−	+
7	2.7.4.8	*Gmk*	−	+	+	+	−	+	+	−	−	+	−	−	+	−
8	2.7.4.9	*Tmk*	+	+	+	+	−	−	−	+	+	+	+	−	+	−
9	4.1.1.65	*Psd*	−	−	+	−	−	+	+	+	+	−	−	−	−	−

*Vc: Vibrio cholera N116961, Bs: Bacillus subtilis 168, Ec: E. coli MG1655, Hi: Haemophilus influenzae Rd KW20, Sp: Streptococcus pneumoniae, Fn: Francisella tularensis sub spp novicida U112, Ab: Acinetobacter baylyi ADP1, Pa: Pseudomonas aeruginosa UCBPP-PA14, Se: Salmonella enterica serv Typhi, Sa: Staphylococcus aureus N315, Mg: Mycoplasma genitalium G37, Mt: Mycobacterium tuberculosis H37Rv, Mp: Mycoplasma pulmonis UAB CTIP and Hp: Helicobacter pylori 26695. “+” Indicates as essential, “− ” Indicates as non-essential.*

The development of antibiotics only against Category A bioterrorism agents may not be economically feasible. An alternative solution would be the development of combinatorial treatment protocols using current antibiotics that are used to treat common bacterial infections. A potential approach toward this goal is to combine the use of common antibiotics already used to treat *B. anthracis*, *Y. pestis*, or *F. tularensis* caused infections with the use of drugs that target enzymes unconditionally essential in all strains of Category A bioterrorism agents. Toward this end, we examined if any of the nine shared essential enzymes have been targeted by existing antibiotics. We found that the FolC (dihydrofolate and tetrahydrofolate biosynthesis pathway), which has two distinct enzymatic activities in some bacteria [27], and is targeted by two antimicrobials, trimethoprim and Rab1 (Fig. 1). Trimethoprim can block dihydrofolate reductase enzymatic activity directly, and its folylpolyglutamate synthetase activity indirectly through the accumulation of a potent inhibitor, dihydrofolate [28]. However, *in vivo* it is only effective against *Y. pestis* [29], while *B. anthracis* and *F. tularensis* are fully and partially resistant against it, respectively [30]. In contrast, Rab1 blocks the enzyme's activity and is active against all three category A bioterrorism agents as well as against both methicillin sensitive and resistant *S. aureus* strains [31].

## Discussion

The treatment and/or prevention of infections with Category A bioterrorism agents in case of an epidemic or deliberate outbreak remains challenging [2]. To identify new targets that may feed the antimicrobial discovery pipeline, we used genome annotations and computational methods to identify genes that encode essential metabolic enzymes in Category A bioterrorism agents. For each of the genus we used several geographical isolates. Although genome variation is generally under 10% across each of the species, their metabolic architecture is different. We identified several single essential enzymes common to all the organisms examined in this study. Comparative genomics and metabolic reconstructions provide a comprehensive understanding of the biology of very closely related organisms. Coupled with flux balance computations these metabolic reconstructions allow the identification of essential metabolic reactions necessary for growth (biomass production) and allow the discovery of novel metabolic targets for potential drug development. We thus conclude that computational identification of single essential enzymes in geographically distinct isolates across several genera is a rapid and cost effective approach for putative antimicrobial target identification.

The identified nine common essential enzymes in the three Category A organisms are shared among several metabolic pathways, but three of them are clustered in the dihydrofolate and tetrahydrofolate biosynthesis pathway that provide the only target (FolC: dihydrofolate synthase) that is affected by existing antimicrobials (Fig. 1). The other enzymes catalyze steps in the coenzyme A, cell wall, and phospholipid metabolism pathways, and nucleic acid subsystem. Of these, thymidylate kinase, which is known to catalyze the conversion of dTMP to dTDP, is essential for the viability of *E. coli* [25] and has been considered as a drug target in other bacteria. Interestingly, in contrast to that seen in *E. coli* [16], [17] or *S. aureus* [19], none of the fatty acid metabolic enzymes were identified as essential in any of the three genera.

Of note, these common essential enzymes can only be viewed as potential targets for at least two reasons. First, they should display sufficient structural similarity in all strains, especially in their catalytic sites, so a common inhibitor can potentially be developed against them. Secondly, their human orthologs should have sufficient dissimilarity and/or not be essential in order to avoid potential toxicity [3]. Finally, none of these targets should be considered for monotherapy, as such approaches would inevitably lead to acquired drug resistance through target mutations. Instead, their inhibition in conjunction with existing strain, or genus-specific antibiotics could form the basis of one potential approach for first line, effective combinatorial therapy of intentional or accidental Category A bioterrorism agent challenge. The simultaneous use of Rab 1 (Fig. 1) with common antibiotics is already being used to treat *B. anthracis*, *Y. pestis*, or *F. tularensis* represents on such combinatorial therapy.

## Materials and Methods

### Metabolic reconstructions

For comparative metabolic analyses we included eight *B. anthracis*, seven *F. tularemia*, and four *Y. pestis* strains. These genomes, which were fully sequenced and analyzed by various research groups provided input data on which we performed the annotations and metabolic reconstruction using the ERGO bioinformatics suite [32]. The genomes with NCBI Bioproject codes along with ORFs, reactions and other metabolite characteristics are provided in Table 1.

We used the updated KEGG ligand/reaction database (http://www.genome.jp/kegg/ ligand.html) to identify all the metabolic reactions in the genomes. Briefly, ORF callings, which were originally (by the sequencing organizations) performed by using either GLIMMER or CRICITA, were imported into the ERGO schema of annotations and pathway analysis. The protein similarities were computed by BLAST ‘all against all’ with over 8.1 million protein sequences being present in the non-redundant ERGO database for over 2,232 genomes [32]. The functional pathways in ERGO are grouped into metabolic and non-metabolic systems that are interconnected into a metabolic network between subsystems, such as amino-acids, carbohydrates, lipids, secondary metabolism, sulfur and phosphorus metabolism, etc. The non-metabolic pathways include virulence, secretion, drug resistance, pro-phages, etc. In case of missing steps within a given pathway, we searched for orthologs or published experimental evidence and gap filled the missing steps. The functional role of the enzymes with complete or incomplete Enzyme Commission (EC) number were identified from the functional categories present in the ERGO genome analysis suite.

The associated biochemical reactions for each of the enzymes were selected from the KEGG reaction database (http://www.genome.jp/kegg/ligand.html). The metabolic reactions were classified into three categories: cellular (reactions in the cytoplasm), transport reactions (involving both the intra and extracellular metabolites), and exchange reactions (either uptake or excretion metabolites) similar to our recent studies in *S. aureus* [19]. The biochemical compounds/reactions that do not have transport systems, or those without experimental evidence in *B. anthracis*, *F. tularensis*, or *Y. pestis* were considered “unlikely reactions” and were excluded from the flux balance analysis (FBA) computations. Individual transport reactions were added from the ERGO pathway collection. All the reactions and their corresponding KEGG identifiers (reaction IDs and compound IDs) were used in the FBA computations [17], [19].

### Flux balance analyses

The stoichiometry of each of the metabolic reactions was adopted from the KEGG ligand database and ERGO bioinformatics suite. FBA were computed similar to methodology used for *S. aureus* [19] and of *E. coli* [16], [17]. As there is no measured or predicted biomass composition published for *F. tularensis*, for this organism we used the biomass components of Gram-negative *E. coli* MG1655 strain [15], [16]. Similarly, for *B. anthracis*, we used a close relative Gram-positive *Bacillus subtilis* strain 168 [33] and for *Y. pestis*, we again used *E. coli* MG1655 biomass components [15], [16], [17], [18].

For essentiality test, we used the mapping between genes and EC numbers. A gene may be mapped to multiple EC numbers (catalyzing multiple reactions) and an EC number may be mapped to multiple genes (having multiple genes catalyzing the same reaction). Accordingly, gene deletion may disable multiple reactions associated with multiple EC numbers or no reaction (when multiple genes are associated with the reaction). We used Omnigraffle (http://www.omnigroup.com/products/omnigraffle/) for generating network diagrams. We used open source software Gephi (http://gephi.org/) to arrange the nodes and used modified perl/python scripts for data analysis and extraction to create svg format files.

### Metabolite essentiality

A metabolite is considered as being essential when the cell cannot produce biomass when all the reactions that consume the metabolite are silenced, i.e., a metabolite is considered essential if the cell cannot produce biomass without the availability of that metabolite. To obtain only feasible targets, we only identify metabolites that have two to five consuming reactions (most metabolites that have only one consuming reaction is captured in our single essential prediction). In addition, we remove the metabolites that belong to the biomass components. The essential genes were compared to the experimentally identified essentials using the DEG database [24].

## Supporting Information

Table S1 **Metabolic essential enzymes and their gene identifiers of the individual strains of Category A pathogens.**
(XLS)
